# Synthesis and Characterisation of Nanocrystalline Co_x_Fe_1−x_GDC Powders as a Functional Anode Material for the Solid Oxide Fuel Cell

**DOI:** 10.3390/ma17153864

**Published:** 2024-08-04

**Authors:** Laura Quinlan, Talia Brooks, Nasrin Ghaemi, Harvey Arellano-Garcia, Maryam Irandoost, Fariborz Sharifianjazi, Bahman Amini Horri

**Affiliations:** 1 School of Chemistry and Chemical Engineering, Faculty of Engineering & Physical Sciences, University of Surrey, Guildford GU2 7XH, UK; 2 Department of Process and Plant Technology, Brandenburg University of Technology (BTU) Cottbus-Senftenberg, 03046 Cottbus, Germany; arellano@b-tu.de; 3 Department of Materials and Metallurgical Engineering, Amirkabir University of Technology, Tehran 15916-34311, Iran; 4 Center of Advanced Materials and Structures, School of Science and Technology, The University of Georgia, Tbilisi 0159, Georgia

**Keywords:** Co_x_Fe_1-x_GDC powders, SOFC anode, solid oxide fuel cell, electrical conductivity, SOFC materials

## Abstract

The necessity for high operational temperatures presents a considerable obstacle to the commercial viability of solid oxide fuel cells (SOFCs). The introduction of active co-dopant ions to polycrystalline solid structures can directly impact the physiochemical and electrical properties of the resulting composites including crystallite size, lattice parameters, ionic and electronic conductivity, sinterability, and mechanical strength. This study proposes cobalt–iron-substituted gadolinium-doped ceria (Co_x_Fe_1-x_GDC) as an innovative, nickel-free anode composite for developing ceramic fuel cells. A new co-precipitation technique using ammonium tartrate as the precipitant in a multi-cationic solution with Co^2+^, Gd^3+^, Fe^3+^, and Ce^3+^ ions was utilized. The physicochemical and morphological characteristics of the synthesized samples were systematically analysed using a comprehensive set of techniques, including DSC/TGA for a thermal analysis, XRD for a crystallographic analysis, SEM/EDX for a morphological and elemental analysis, FT-IR for a chemical bonding analysis, and Raman spectroscopy for a vibrational analysis. The morphological analysis, SEM, showed the formation of nanoparticles (≤15 nm), which corresponded well with the crystal size determined by the XRD analysis, which was within the range of ≤10 nm. The fabrication of single SOFC bilayers occurred within an electrolyte-supported structure, with the use of the GDC as the electrolyte layer and the CoO–Fe_2_O_3_/GDC composite as the anode. SEM imaging and the EIS analysis were utilized to examine the fabricated symmetrical cells.

## 1. Introduction

Solid oxide fuel cells (SOFCs) are versatile and efficient power generation technology that converts fuel’s chemical energy into electricity with an impressive efficiency of over 60% [1,2,3]. Typically, SOFCs employ yttria-stabilized zirconia (YSZ) as the electrolyte due to its significant ionic conductivity at high temperatures ranging from 800 °C to 1000 °C [4]. While this enables the use of various fuels, including biogas, natural gas, syngas, hydrogen, and solid carbon, operating at such high temperatures can lead to interfacial diffusion, electrode sintering, thermal and mechanical instability, and high manufacturing and maintenance costs [5]. Lowering the operational temperature is a commonly proposed method to mitigate cell degradation rates and facilitate the utilisation of more affordable materials and fabrication techniques [6]. However, running SOFCs at temperatures lower than 600 °C can significantly impact electrode reaction kinetics, reduce oxide ions’ mobility in the electrolyte, and diminish the internal reforming reaction rate [7]. The process of internally reforming hydrocarbons becomes especially demanding in low-temperature SOFCs due to the endothermic properties of the steam-reforming reaction. This reaction can experience notable deceleration when operating temperatures are decreased [8]. Operating at temperatures lower than 700 °C thermodynamically favours carbon deposition processes in the anode, further complicating the fuel cell operation [9].

Nickel-based anodes, such as Ni-YSZ, are used in SOFCs due to their compatibility with other cell components, effective catalytic activity for hydrogen oxidation, and excellent electrical conductivity [10]. However, Ni is prone to hydrocarbon cracking, leading to significant carbon deposition on its surface, and the anode structure experiences dimensional changes while undergoing a redox behaviour [11]. Therefore, carefully selecting the material used as an electrocatalyst for the anode electrode is crucial, particularly at lower operating temperatures. To address these challenges, researchers have developed an approach that involves partially substituting Ni with a reforming catalyst in the fuel electrode. The catalyst can be composed of noble metals like Ru, Rh, and Pt or transition metals like Fe, Cu, and Co. [12,13,14]. Unfortunately, the excellent resistance of noble metals to carbon deposition is overshadowed by their scarcity and high cost, limiting their application [15,16]. However, partially substituting Ni with elements like Co and Cu has effectively reduced coke formation and offered a more economically feasible option. Co has emerged as a favourable anode material for SOFCs due to its similar catalytic properties to Ni but with a significantly lower tendency for coking [17]. Incorporating varying amounts of Co in Ni-based anodes has yielded favourable results in terms of microstructure and catalytic activity, leading to enhanced cell performance at lower temperatures. Ni-Co alloys offer excellent electrochemical activity and thermal stability thanks to Co’s highly compatible melting point. Moreover, Co’s superior oxidation resistance compared to Ni enables effective cell functioning at lower voltages, making it highly desirable for SOFCs operating with high-hydrocarbon fuels [18]. Recent research has shown that bimetallic Ni-Fe alloys outperform pure Ni anodes in SOFCs [19]. Anodes containing 10 wt.% Fe have been found to exhibit the highest catalytic activity due to their minimal overpotential [20]. Furthermore, the incorporation of Fe in Ni anodes significantly reduces carbon deposition due to Fe’s lower carbon formation activity compared to Ni [21]. In the past few years, there has been a surge of interest in gadolinium-doped ceria (GDC) composites as highly promising candidates for the anode in SOFCs [22]. GDC is commonly regarded as a viable alternative to YSZ for the fuel electrode. It is well-established that Ni-GDC exhibits superior resistance to carbon deposition compared to conventional Ni-YSZ. In a reducing atmosphere, GDC functions as a mixed conductor of ions and electrons, demonstrating enhanced ionic conductivity compared to YSZ, particularly at lower temperatures [23].

The active sites for electrochemical reactions in Ni-YSZ and Ni-GDC anodes are typically attributed to the triple-phase boundary (TPB), which forms at the intersection of pores and ionic and electronic conductors. It is noteworthy that in the case of Ni-GDC composite cermet, the electrochemical reaction is not solely influenced by the TPBs but also by the presence of double-phase boundaries (DPBs) between the pores and GDC [24].

This exciting factor holds tremendous potential for enhancing the performance of anodes in SOFCs. Despite extensive research to enhance the performance and stability of Ni-GDC anodes, the complete elimination of Ni-related degradation phenomena remains a significant challenge. Hence, there is still a need for high-performance anode materials to overcome the limitations of Ni-based anodes. The purpose of this study is to create a novel Ni-free anode nanocomposite utilising GDC as the base material. To achieve this, Co and Fe were selected as the catalytic components to achieve comparable activity levels suitable for low-temperature SOFCs. To this end, nanocrystalline anode composites of Co_x_Fe_1-x_GDC (x = 0.20, 0.50, and 0.80) were synthesised through the co-precipitation method. Ammonium tartrate, a cost-effective and environmentally friendly precipitant, was employed in the synthesis process. The synthesised powders were characterised for their physiochemical properties and catalytic activity using various techniques such as Fourier transform infrared spectroscopy (FTIR), simultaneous thermogravimetric and differential scanning calorimetry analysis (DSC-TGA), X-ray powder diffraction (XRD), Raman spectroscopy, field emission scanning electron microscopy (FESEM), and Energy Dispersive X-ray Spectroscopy (EDS). Electrolyte-supported symmetrical cells were subsequently fabricated and investigated through the utilisation of electrochemical impedance spectroscopy (EIS) and scanning electron microscopy (SEM). The EIS data were evaluated using Zview software (accessed in September 2021).

## 2. Materials and Methods

### 2.1. Material

The precursors used for the synthesis of CO–Fe/GDC anode samples included gadolinium nitrate hexahydrate iron nitrate nonahydrate (Fe(NO_3_)_3_·9H_2_O, 99.0%, Merck, Rahway, NJ, USA), (Gd (NO_3_)_3_·6H_2_O, 99.9%, Sigma-Aldrich, St. Louis, MO, USA), cerium nitrate hexahydrate (Ce(NO_3_)_3_·6H_2_O, 99%, Sigma-Aldrich), cobalt nitrate hexahydrate (Co(NO_3_)_2_·6H_2_O, 99.0%, Merck), and ammonium tartrate ((NH_4_)_2_C_4_H_4_O_6_, 98%, Sigma-Aldrich). Polyvinylpyrrolidone (PVP) was utilised as a dispersant procured from Sigma-Aldrich. Graphite (TB-17 MTI) and carbon nanopowder (particle size less than 100 nm, Sigma-Aldrich) served as a pore-forming agent, and α-terpineol (C_10_H_18_O, Supelco, Bellefonte, PA, USA, analytical grade) and ethanol (>99.8%, Merck) were the chosen solvents. The electrolyte layer was added via vacuum filtration of a solution containing 10 mol% gadolinium doped cerium (IV) oxide (GDC_20_, Sigma-Aldrich) suspended in 2-Propanol (99.9%, Sigma-Aldrich).

### 2.2. Powder Synthesis

**Figure 1:**
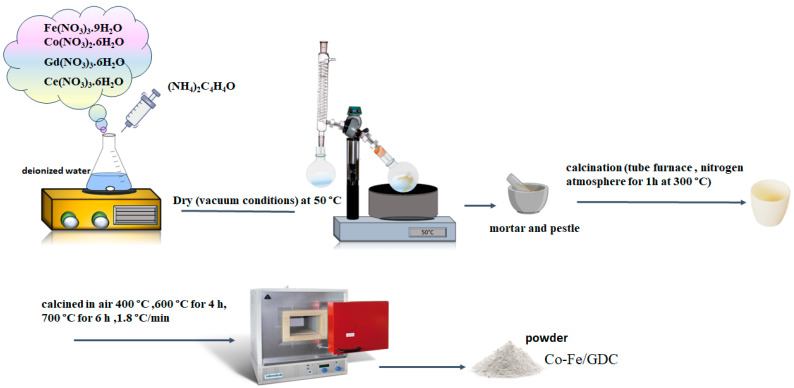
The synthesis process of the Co-Fe/GDC powder.

Six anode samples were prepared using a nanopowder of Co_x_Fe_1−x_-Gd_0.1_Ce_0.9_O_1.95_, resulting in a total of six Co-Fe/GDC anode samples. The samples had varying fractions of x, namely 0.2, 0.5, and 0.8, representing Co-Fe volume ratios of 4:1, 1:1, and 1:4, respectively. The first batch of samples, consisting of Co_x_Fe_1-x_GDC (x = 0.2, 0.5, and 0.8) with a bimetal/GDC ratio of 50/50 (Vol. %), were labelled as S1, S2, and S3, respectively. The second batch, named S4, S5, and S6, had the composition of Co_x_Fe_1-x_GDC (x = 0.2, 0.5, and 0.8) with a bimetal/GDC ratio of 60/40 (Vol. %). In summary, the dissolution process involved adding the stoichiometric quantities of Gd (NO_3_)_3_·6H_2_O, Ce (NO_3_)_3_·6H_2_O, Fe (NO_3_)_3_·9H_2_O, and Co (NO_3_)_2_·6H_2_O compounds into deionised water. Each combination solution, at a concentration of 0.18 M, was added drop by drop to an aqueous solution of (NH4)_2_C_4_H_4_O (with an excess of 2.0%) and stirred at room temperature. After 4.0 h, the precipitates were dried under a rotary evaporator coupled to a vacuum pump at 50 °C. The dried sample was ground using an agate pestle and mortar before undergoing calcination. Initially, calcination was performed in a tube furnace under a nitrogen atmosphere for 1 h at 300 °C. Subsequently, the samples were calcined at 400 °C or 600 °C for a duration of 4.0 h in the air, with a heating rate of 1.8 °C/min. The choice of using nitrogen and specific calcination temperatures was determined simultaneously (DSC-TGA). Further calcination was performed for the sample being used in the cell fabrication based on the X-ray diffraction analysis results. The final calcination method occurred in a muffle furnace at 700 °C in the air for 6.0 h, utilising a rate of 1.8 °C/min. Figure 1 schematically shows the processing steps involved in synthesising Co-Fe/GDC samples.

### 2.3. Cell Fabrication

**Figure 2:**
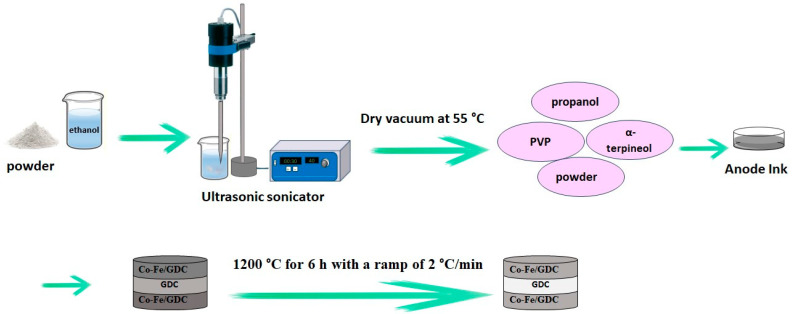
The fabrication of a symmetrical cell.

Suspension inks of S2 and S5 samples with an equal volume ratio of Co-Fe/GDC (50:50 vol.%) (S_2_) and Co-Fe/GDC (60:40 vol.%) (S_5_) were prepared for electrochemical studies. To achieve the required porosity for the anode involved dispersing specific amounts of ceramic powder in ethanol and subjecting it to ultrasonication using a FB-705 sonicator (Fisher Scientific, Waltham, MA, USA) at 40 amps with 5 s pulses for a total active time of 30 min, followed by the addition of pore formers (1.0 wt.% nano-sized carbon and 5.0 wt.% macro-sized carbon) and sonication for further 5.0 min. Afterward, the resulting mixture was dried under a vacuum at 55 °C. The powders obtained were crushed in an agate mortar and subsequently utilised to create ink. The powders were evenly dispersed in a combination of propanol and α-terpineol solvents (in a 50:50 ratio by volume), with the inclusion of 3% PVP as a polymer-based dispersing agent. Anode and electrolyte pellets were prepared using an MTI Corporation YLJ-24T desk-top (MTI Corporation, Richmond, VA, USA) manual pressing machine, exerting a force of 5.0 MPa for 30 s, followed by an increase to 10 MPa for an additional 90 s. The GDC pellets were pre-sintered in a furnace, in the air at 950 °C for 4.0 h, with a slow ramp of 1.5 °C/min to prevent thermal stress and avoid breaking the disks. The anode ink was deposited on both sides of GDC using screen printing to achieve the desired thickness for the symmetrical cell. The fabricated cell was then co-sintered at 1200 °C for 6.0 h at a rate of 2 °C/min. The process of fabricating symmetric cells is represented in Figure 2, showcasing the various steps.

### 2.4. Characterisation

A DSC-TGA analysis was conducted using an SDT Q600 instrument (TA Instruments, New Castle, DE, USA) to examine the thermal decomposition of the dried powders. The samples underwent a slow heating process in the presence of air, beginning at room temperature and steadily increasing to 900 °C at a rate of 5 °C/min. Additionally, to ensure the absence of carbon at the desired final calcination temperature, the DSC-TGA analysis of the calcined samples at 700 °C was carried out using the same experimental conditions.

FT-IR spectroscopy was employed to examine the bond characteristics of the samples before and after calcination at temperatures of 400 °C, 600 °C, and 700 °C. The spectra were acquired by scanning within the range of 525–4000 cm^−1^, with a resolution of 4.0 cm^−1^ (recorded at an average of 65 scans). The Agilent Cary 640 Fourier transform infrared (FTIR) spectrometer was utilised to record the infrared spectra of the samples. The specimens for the FTIR analysis were prepared in transmittance mode utilising the KBr pellet technique. Initially, the Co-Fe/GDC beads were ground into powder, then combined with KBr, and subsequently placed in an oven at 80 °C to reduce the moisture content of the specimens.

An XRD analysis was used to identify the crystalline phase in the synthesised powder materials, both pre- and post-calcination. The XRD analyses were performed utilising a PANalytical X′Pert 3 Powder diffractometer (Malvern Panalytical Ltd., Malvern, United Kingdom). The Cu-Kα radiation had a wavelength of 1.5418 Å, within the 2θ angle (10–90°) at 30 mA and 40 kV. To calculate the crystal size, the Scherrer formula (Equation (1)) was utilised using data obtained from the XRD patterns, and the analysis was performed using High Score Plus (Malvern Panalytical Ltd., Malvern, UK, HighScore 5.1) and Origin (OriginLab, Newton, MA, USA, Origin 9.9) software.

*D* = 0.9*λ*/*βcosθ* (1)

In the equation, *λ* denotes the X-ray wavelength in nm, the full width at half maximum intensity (FWHM) in radians is denoted by the symbol *β*, the crystal size in nm is denoted by *D*, and the Bragg angle in radians is indicated by θ [25].

The estimation of the lattice parameter (*α*) was conducted by applying the Bragg equation (Equation (2)) [26]:

*λ*/(2sin*θ*) = *α*/(*h*^2^ + *k*^2^ + *l*^2^)^1/2^ (2)

where *h*, *k*, and *l* represent the Miller indices of the crystal lattice parameters.

The composition of the anode material was further confirmed through Raman spectroscopy. Specifically, a Renishaw 2000 with a 514 nm green laser and 40 mW power was utilised within the range between 250 and 700 cm^−1^. The samples underwent an elemental analysis using an EDX microanalysis (JEOL, Tokyo, Japan). The instrument employed for this analysis was a JEOL JSM-7100F (JEOL, Tokyo, Japan) thermal field-emission electron microscope equipped with an EDX detector, operating at an acceleration voltage of 15.0 kV. The anode samples were analysed for their microstructure using an SEM analysis. A Hitachi HD-2300A STEM (Hitachi High-Tech Corporation, Tokyo, Japan) at 200 kV, was used to capture SEM images of the samples, which were carefully placed on carbon tape.

The electrochemical tests were conducted in an environment containing H2 with a 70 mL/min flow rate. The EIS analysis was employed to examine the electrochemical characteristics of the constructed symmetrical cell, and a potentiostat-galvanostat analyser (Interface 1010E, Gamry, Philadelphia, PA, USA) was utilised. The frequency spectrum ranged from 0.1 Hz to 2 MHz, and an AC voltage amplitude of 10 mV was applied. The experiments were conducted at 450 °C–650 °C. The impedance spectra were evaluated using the equivalent circuit provided by the Zview software program.

Area-specific resistance (ASR) was calculated using Equation (3):

ASR = R_pt_ × A/2 (3)

The calculation of the polarisation resistance (R_pt_) for a given area of the apparent surface of a single electrode (A) can be obtained from Equation (4) as follows [27]:

R_pt_ = (R_p1_ + R_p2_)/2 (4)

In this equation, R_pt_ represents the interfacial anode and electrolyte polarisation resistance. R_p1_ represents the high-frequency arc linked to the charge transfer mechanism through the anode composite and at the anode/GDC electrolyte interface. R_p2_ denotes the low-frequency arc associated with the diffusion process at the anode surface. A is the active area of the anode (effective current collector area). In this study, the composite anode was consistently printed in a circular shape with a radius of 0.4 cm, resulting in a total area of 0.6 cm^2^ for all symmetrical cells.

## 3. Results and Discussion

### 3.1. Simultaneous DSC/TGA Analysis

**Figure 3:**
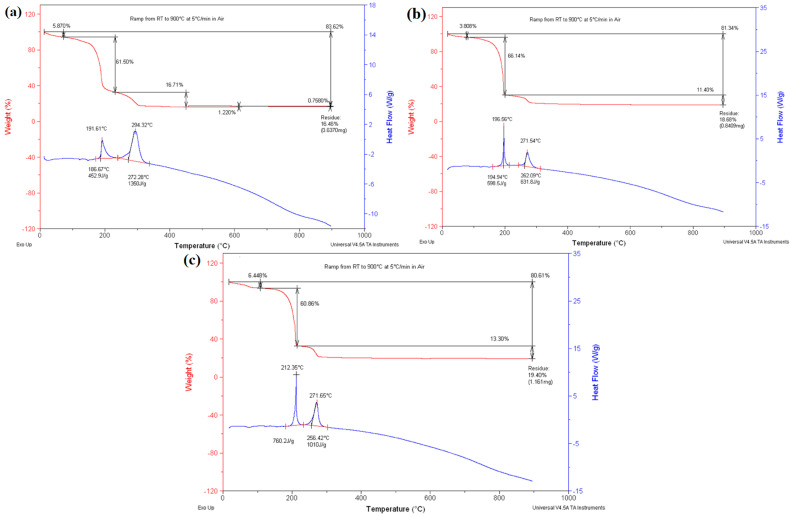
DCS-TGA profiles of the thermal decomposition of the prepared samples, including (**a**) S_1_, (**b**) S2, and (**c**) S_3_ before calcination.

The TGA results for the samples S_1_, S_2_, and S_3_ with the compositions of Co_x_Fe_1-x_GDC (x = 0.2, 0.5, and 0.8) and the Co-Fe-to-GDC volume ratio of 50/50, before calcination, are shown in Figure 3. The initial weight loss can be ascribed to the evaporation of any residual water during the heating phase from ambient temperature to 150 °C. The weight losses were relatively low, with values of 5.87%, 3.81%, and 6.45% observed for samples S1, S2, and S3, respectively. The second and main weight loss, accounting for 61.5%, 66.1%, and 60.9% of the total weight loss in S_1_, S_2_, and S_3_, respectively, was due to the removal of ammonium nitrate, which was the main by-product of the reaction, taking place at 150–200 °C. The exothermic nature of this process was evident from the heat flow peak observed at approximately 200 °C in all samples, which was consistent with the explosive properties of ammonium nitrate. The final stage of weight loss, occurring between 200 °C and 350 °C, accounted for 16.7%, 11.4%, and 13.3% of the total weight loss in S_1_, S_2_, and S_3_, respectively. This weight loss was due to the elimination of any remaining carbon content and the conversion of metal tartrates into metal oxides (FeO/Fe_2_O_3_, CoO, CeO_2_, and Gd_2_O_3_) [28,29]. The weight loss observed during the metal tartrates’ decomposition and the oxidation was primarily due to the elimination of carbonaceous materials, including carbon monoxide and dioxide. It is crucial to mention that no significant further weight loss was observed after reaching a temperature of approximately 400 °C. Moreover, the absence of any subsequent changes in the heat flow indicated that complete calcination could be achieved above this temperature threshold.

Upon calcination at 400 °C–600 °C, the calcined samples showed the existence of minor quantities of carbon. Compared to the TGA result of the calcined sample at 600 °C, there was a significant 2.971% decrease in weight at 400 °C, indicating a higher presence of carbonaceous material. The results aligned with the findings from the EDX analysis. The carbon percentage decreased as the calcination temperature rose. It is worth mentioning that the TGA-DSC of S_1_ showed a slight increase in weight, considering that S_1_ contained the highest iron content among the three compositions. The increased weight in TGA may be attributed to the buoyant effect of air [30]. Additionally, a different theory posits that the ferromagnetism of the FeO present in the samples could be responsible. FeO has been confirmed to exhibit ferromagnetic properties, and extensive studies have been conducted on its characteristics [31]. The samples displayed magnetism when magnetic stirrers were attracted to them.

**Figure 4:**
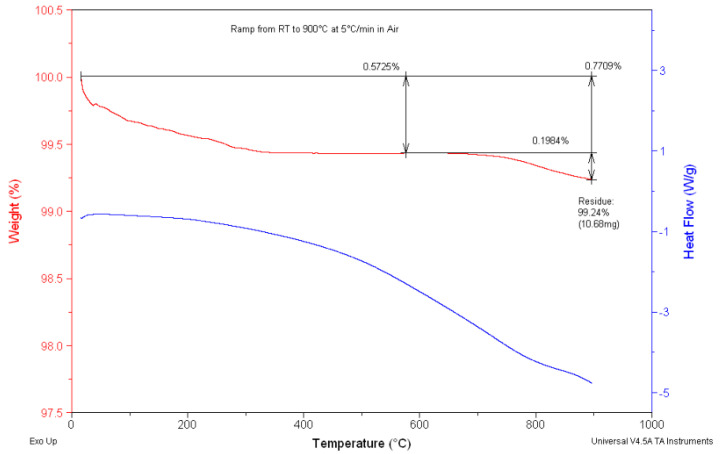
DCS-TGA curve of the thermal decomposition of S_2_ after calcination at 700 °C for 6 h in air.

To examine the existence of carbon in the samples employed for the fabrication of the cells, a DSC-TGA analysis was performed after the calcination process at 700 °C (Figure 4). According to the EDX data corroborating the TGA findings, only a minimal presence of carbon was left in the sample after 700 °C, affirming that 700 °C was the optimum calcination temperature. Chen et al. [32] emphasised the significance of accurate baseline calibration, as discrepancies stemming from varying experimental parameters or thermal masses can result in non-linear baselines. The curly baseline observed in the DSC curves can be attributed to changes in the sample’s heat capacity during thermal processes.

**Figure 5:**
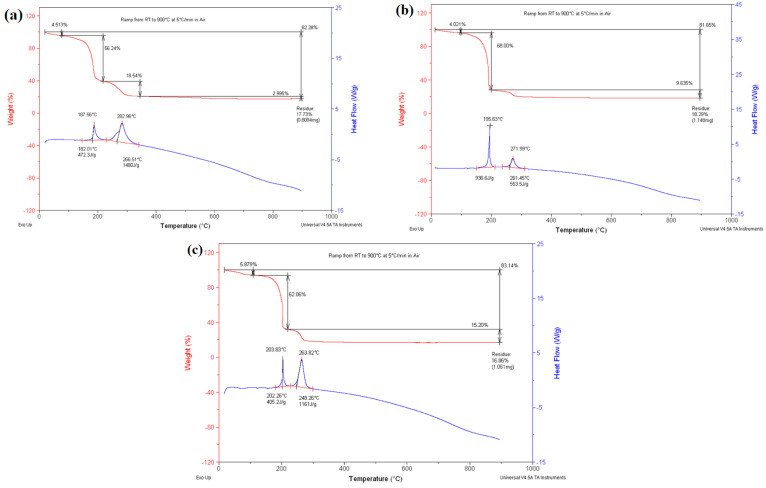
DSC-TGA profiles of the thermal decomposition of the prepared samples, including (**a**) S_4_ (**b**) S_5_, and (**c**) S_6_ before calcination.

TGA curves for the synthesised Co_x_Fe_1-x_O/GDC with the composition of x = 0.2, 0.5, and 0.8 and the Co-Fe-to-GDC volume ratio of 60/40 named S_4_, S_5_, and S_6_, prior to calcination, can be seen in Figure 5. The samples underwent thermal decomposition in three distinct steps. Between approximately room temperature and 100 °C, S4, S5, and S6 experienced a minor weight loss of 4–5%. This can be attributed to the extraction of water from the crystals. [33]. The second and main weight loss occurred between 100 °C and 220 °C, averaging 62.10% across the samples due to the combustion of the ammonium nitrate present in the samples. This was confirmed by the melting point of ammonium nitrate being at 166 °C [28], as the sharp decrease in weight began after that temperature. The subsequent weight loss that occurred afterwards was due to the breakdown of organic compounds in the samples [34]. The initial conversion of the metal tartrates to metal oxides was observed by the corresponding DCS peaks between 185 °C and 205 °C, indicating an exothermic reaction [33]. A further breakdown of the remaining organic matter and the conversion of the metal tartrates occurred between 220 °C and 350 °C, resulting in weight losses of 18.54%, 9.63%, and 15.20 for S_4_, S_5_, and S_6_, respectively. Although this was more gradual than the weight loss associated with ammonium nitrate, it was still significantly sharper than the weight loss related to the removal of water due to the combustion of organic compounds at that temperature [34]. The exothermic DCS peak between 260 °C and 285 °C confirmed the decomposition of all metal nitrates present, as confirmed by the literature [27,33,34].

According to the DSC-TGA results, no further weight loss was observed after the temperature reached 300 °C. Furthermore, the absence of any subsequent changes in heat flow suggested that complete calcination could be accomplished above 300 °C. Both 400 °C and 600 °C were chosen to compare the impurities removed and crystal size in different calcination temperatures. It is preferable to have a small crystalline size with low impurities; higher temperatures, which are required to remove more impurities, result in larger crystal sizes.

**Figure 6:**
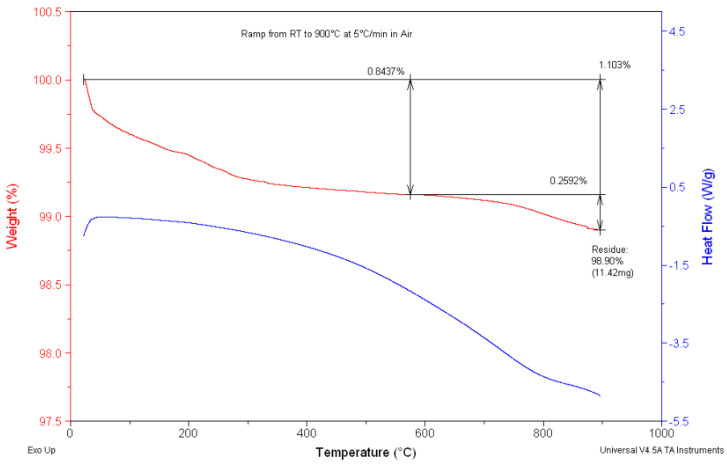
DCS-TGA curve of the thermal decomposition of S5 after calcination at 700 °C for 6 h in air.

Figure 6 shows the TGA analysis for S5 after calcination for 6.0 h at 700 °C. The results confirm that all by-products, including ammonium tartrate, were completely removed and decomposed.

### 3.2. FT-IR Analysis

**Figure 7:**
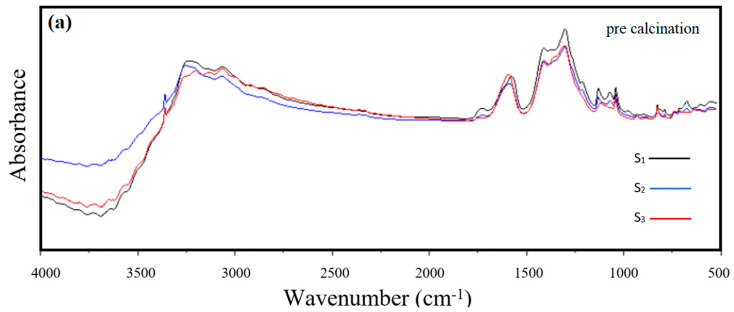

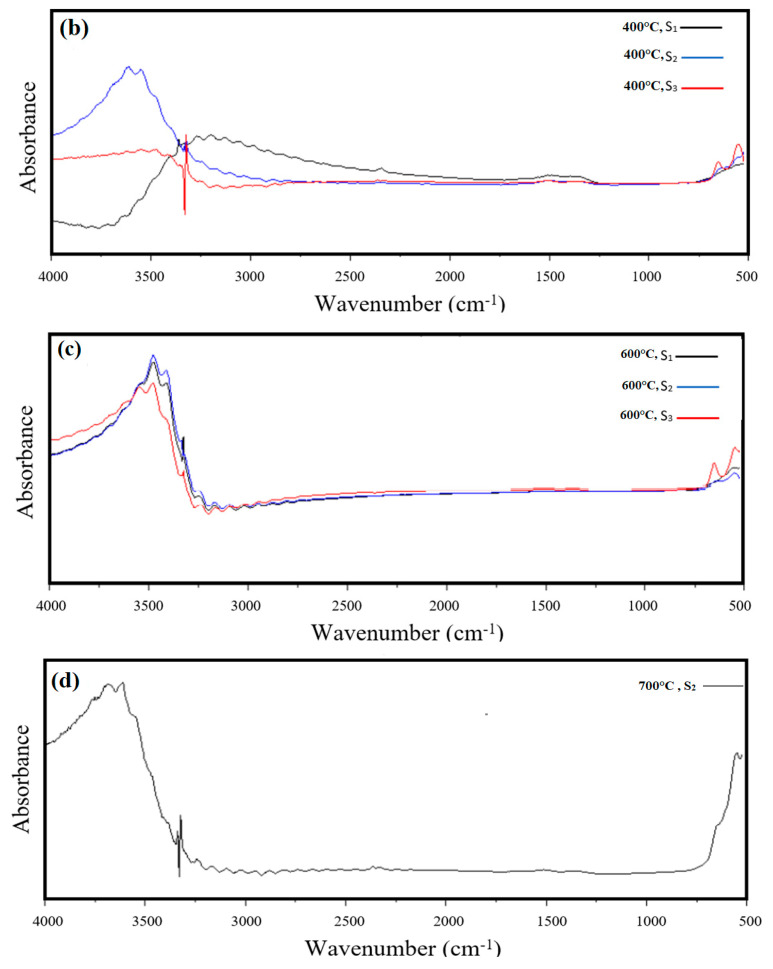
FT-IR Spectra for (**a**) pre-calcination, (**b**) calcination sample S1, S2, and S3 at 400 °C, (**c**) calcination sample S1, S2, and S3 at 600 °C, (**d**) S2 post-calcination at 700 °C.

An FT-IR analysis was performed on samples S_1_, S_2_, and S_3_ both before and after calcination at different temperatures to confirm the characterisation of ceramic materials used for fabricating the symmetrical cell (Figure 7). Before calcination (Figure 7a), the presence of the O-H bond indicated the existence of water in all samples, and the peak of the corresponding was visible within the range of 3000 cm^−1^ and 3500 cm^−1^, expressing the O-H stretching vibration. This observation can be attributed to the fact that when the samples were exposed to the air, they absorbed a small amount of moisture present in the environment [35,36]. It is also possible that a peak corresponding to O-H bond bending may be present at approximately 1650 cm^−1^ [37]. Moreover, the peaks detected within the range of 1000 cm^−1^ to 1800 cm^−1^ are likely due to the presence of C-H bonds, as well as both COO- and –COO bonds found in the tartrate structure, and those associated with the presence of ammonium nitrate in the by-product [38]. The synthesis of the powders took place at room temperature, and it can be inferred that the reaction between tartrate and metal nitrates resulted in the formation of ammonium IV nitrate, which exhibited an orthorhombic crystalline structure [39]. Compared to other phases, ammonium IV nitrate showed broad peaks spanning from 1200 cm^−1^ to 1600 cm^−1^. Such intensity peaks can be observed in Figure 5a for samples prior to calcination [40].

After undergoing calcination at 400 °C (Figure 7b), 600 °C (Figure 7c), and 700 °C (Figure 7d), the FT-IR analysis was used to verify the removal of ammonium nitrate and the conversion of metal tartrates to metal oxides during decomposition and oxidation processes. As shown in Figure 5b, the peaks within the range of approximately 1000 cm^−1^ to 2000 cm^−1^ exhibited a reduced intensity, indicating that the calcination temperatures, even at 400 °C, were adequate for a complete reaction. The FT-IR spectra of all three samples demonstrated comparable peaks before and after calcination, indicating a consistent approach for powder synthesis and calcination, supported by the TGA results. The appearance of minor peaks within the range of 2300 cm^−1^ to 2400 cm^−1^ could be attributed to the presence of CO_2_ gas, which was likely formed through interactions with the surrounding environment. Notably, these peaks were consistently observed in the FT-IR spectra both after and before calcination. The introduction of this CO_2_ could have occurred through something as simple as breathing on the equipment, thereby making its removal a considerably challenging task [41]. The sample contained metal–oxygen bonds, indicated by the peaks at 900 cm^−1^ and 525 cm^−1^. Nevertheless, identifying each metal oxide within the spectra proves difficult. The presence of gadolinium oxides, cerium, cobalt, cerium, and iron was confirmed by subjecting S2 to calcination at 700 °C and subsequently conducting Raman spectroscopy.

**Figure 8:**
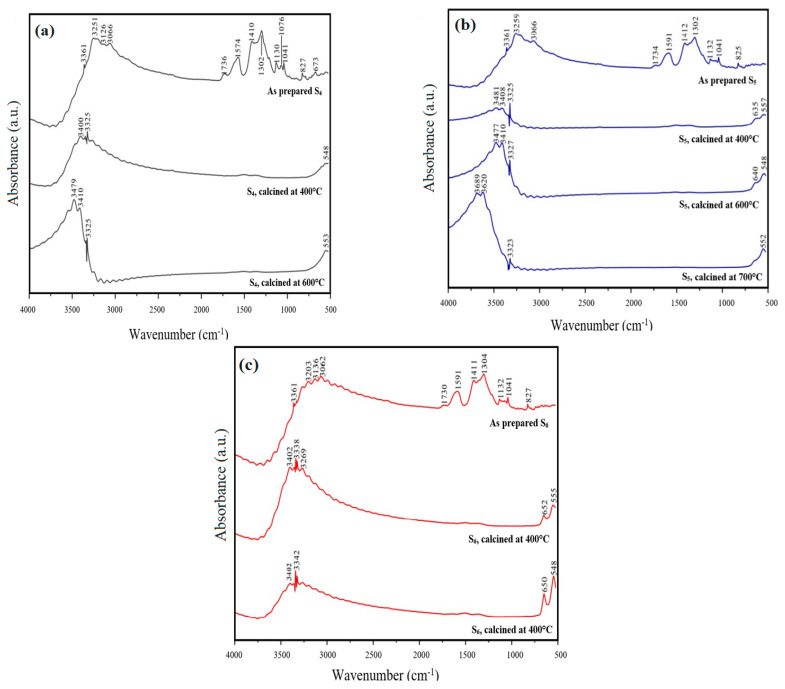
FT-IR spectra of (**a**,**c**) S4 and S6 pre- and post-calcination at 400 °C and 600 °C, (**b**) S5 pre- and post-calcination at 400 °C, 600 °C, and 700 °C.

Figure 8 displays the FT-IR spectra of S_4_, S_5_, and S_6_, each undergoing different calcination temperatures. A prominent feature observed in all samples is a strong, broad peak between 3000 cm^−1^ and 3750 cm^−1^, which signifies the stretching of the O-H bond. This peak is attributed to the presence of adsorbed water within the samples [38]. The DCS-TGA analysis of S_5_ after calcination at 700 °C showed a residual weight loss of 0.8437%, confirming the presence of water in the samples. In the samples prepared, the FT-IR spectra exhibited multiple peaks spanning from 500 cm^−1^ to 1800 cm^−1^. The peaks situated at approximately 1600 cm^−1^ corresponded to the vibration of the -COO bond, while the peaks around 1410 cm^−1^ indicated the stretching of the -COO bond [42]. These peaks solely existed in the pre-calcinated samples since the metal tartrates of Fe, Co, Ce, and Gd were still present. Additionally, all other peaks between 500 cm^−1^ and 1800 cm^−1^ were attributed to the deformation of either NH_4_^+^ or NO_3_^−^, which are present in ammonium nitrate [40]. After calcination at all temperatures, the peaks attributed to the metal tartrates and ammonium nitrate disappeared. Instead, new peaks emerged between 500 cm^−1^ and 650 cm^−1^, representing the stretching of the metal–oxygen bonds. These FT-IR spectra complemented the findings of the XRD analysis, which also confirmed the removal of ammonium tartrate and conversion of metal nitrates to metal oxides after calcination at all temperatures.

### 3.3. XRD Analysis

**Figure 9:**
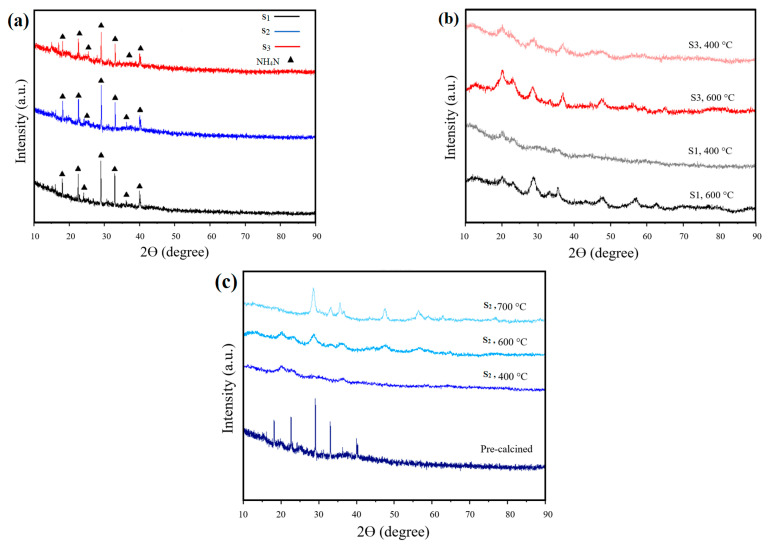
XRD patterns of (**a**) samples before calcination, (**b**) sample S_1_ and S_3_ after calcination at 400 °C and 600 °C, (**c**) S_2_ calcined at different temperatures of 400, 600, and 700 °C.

The XRD analysis was performed on samples S_1_, S_2_, and S_3_ both before and after calcination, and the peaks observed in the XRD analysis were identified in Figure 9. The XRD patterns of the pre-calcined samples are illustrated in Figure 9a, while the profiles after calcination at 400 °C are presented in Figure 9b. Additionally, the XRD analysis was conducted on the S_2_ sample calcined at various temperatures (400, 600, and 700 °C), and the results were compared with the XRD diffraction before calcination, as depicted in Figure 9. For further analysis, the XRD pattern of S_2_ calcined at 700 °C (Figure 9c) was chosen due to the larger crystal size, which allowed for clearer peak identification. This particular powder was also used for the fabrication of the cell, making its composition of utmost importance. The XRD patterns of the pre-calcined samples clearly indicated the existence of ammonium nitrate (Figure 9a). The peaks corresponding to this material dominated the XRD pattern, suggesting its abundance in the samples. During powder synthesis, ammonium tartrate was added in a slight excess (2%) to each metal nitrate as a reactant, resulting in its significant presence and the corresponding high-intensity peaks. These peaks aligned with the ones reported in the literature on the formation of NH_4_NO_3_ at room temperature [40]. The XRD analysis supported the findings from the FT-IR analysis, indicating the removal of ammonium nitrate upon calcination, as demonstrated by the comparison of pre- and post-calcination XRD patterns.

For further details, the analysis of the Scherrer equation’s results is presented in Table 1. The crystal size of the dried samples aligned with our expectations, revealing a size almost 10 times larger than that of the calcined samples. The crystal size observed was nearly 10 times larger than the sizes in the calcined samples. The samples calcined at 400 °C exhibited the smallest crystal sizes, with a slight increase noted as the temperature was raised to 600 °C. Achieving a complete reaction requires a high calcination temperature, but this comes at the expense of smaller crystal sizes. Increasing the calcination temperature to 700 °C was decided upon as a compromise to achieve a larger surface area despite the preference for a smaller size, in accordance with the outcomes of TGAs conducted on the calcined samples, XRD patterns, and EDX analysis. The Braggs equation was employed to analyse the calcined samples lattice parameters, with the findings detailed in Table 2. The outcomes indicated a direct correlation between the lattice parameter and the variation in cobalt concentration, consistent with Vegard’s Law [43].

**Figure 10:**
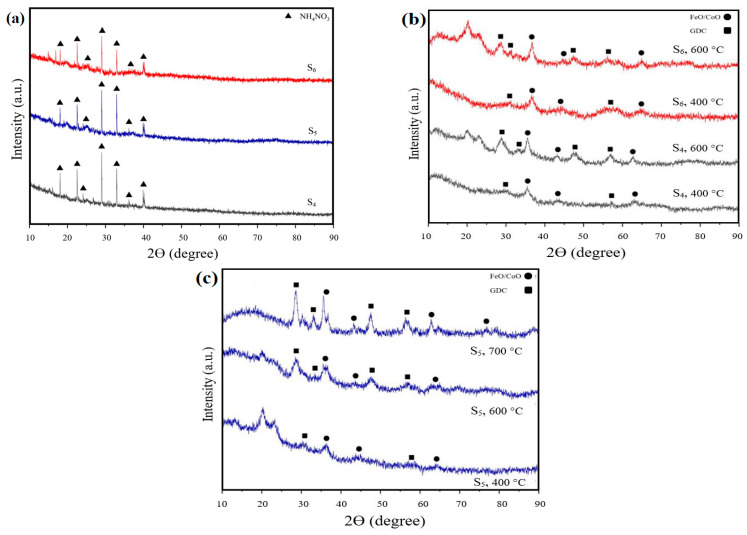
XRD patterns of (**a**) samples before calcination, (**b**) sample S_6_ and S_7_ after calcination at 400 °C and 600°, (**c**) S_5_ calcined at different temperature of 400, 600, and 700 °C.

Figure 10a shows the XRD patterns for as-prepared samples S_4_, S_5_, and S_6_, showing distinct peaks corresponding to ammonium nitrate. The Miller indices and corresponding angles for these peaks are shown in Table 3. The prevalence of peaks for ammonium nitrate was expected in these samples due to the large amount present prior to calcination. Figure 10b,c show the XRD patterns for samples after calcination at different temperatures. The samples calcined at 400 °C did not show clear or distinct peaks and display a significant amount of noise, indicating the presence of amorphous carbon in the samples [44]. The peaks corresponded to FeO, COO, CeO_2,_ and Gd_2_0_3_, with Miller indices and corresponding angles shown in Table 4. Due to the proximity of the XRD peaks for FeO and Coo, and CeO_2_ and Gd_2_O_3_, they were grouped as Co-Fe and GDC on the XRD patterns.

Calcination at 600 °C resulted in XRD patterns with more visible and broad peaks, indicating a small crystalline size. The noisy nature of the XRD patterns suggested the possibility of remaining carbon, so a further calcination process was performed at 700 °C in the presence of air only to ensure the removal of any remaining carbon. In Figure 10b, the XRD pattern after calcination at 700 °C shows an increase in the number of visible peaks, which are narrower, indicating an increase in crystalline size The crystallite size of each sample was computed using the Scherrer equation after the calcination process. The Bragg’s equation was used to find the lattice parameter, and results are presented in Table 5, with all values calculated using the (1 1 1) Co-Fe peak. The findings indicated a correlation between the calcination temperature and the increase in crystalline size, aligning with the existing literature [50]. This also confirmed that noise on the XRD patterns could be partially attributed to the small size of the crystals. Additionally, the lattice parameter of each sample was determined, showing a decrease in lattice parameter with an increase in FeO, following the relationship as outlined by Vegard’s Law. It is generally accepted that lattice contraction is a crucial aspect to consider when assessing lattice parameters, particularly in nanoparticles. Researchers have delved into the effects of lattice contraction on the behaviour of the material [51,52]. The differences in lattice parameters and contraction observed among samples (S1, S2, and S3) were primarily due to their composition (varying iron (Fe) and cobalt (Co) contents). Sample S1 (lowest Co content) showed a modest lattice contraction, increasing slightly from 0.33% at 400 °C to 0.70% at 600 °C, which indicated a minimal lattice distortion. In contrast, sample S2 exhibited a higher initial lattice contraction of 1.99% at 400 °C, which decreased at higher temperatures, which could be due to structural relaxation and defect annealing. Sample S3 (highest Co content) showed the most significant lattice contraction of 2.92% at both 400 °C and 600 °C, indicating substantial lattice distortions, which were not mitigated by increased calcination temperatures. It showed that the increased lattice distortion and contraction in S3 were attributed to the high Co content, which caused significant substitutional stress within the crystal lattice. It can be said that Fe acted as a stabilising agent here, but its concentration was not sufficient to counterbalance the high Co-induced distortions, which can be further studied in the future.

In addition, Kharton et al. [53] studied the effect of particle size on the lattice parameters of GDC nanoparticles. They observed that as the particle size decreased, the lattice parameter also decreased, indicating a lattice contraction in smaller GDC nanoparticles.

### 3.4. Raman Spectroscopy

**Figure 11:**
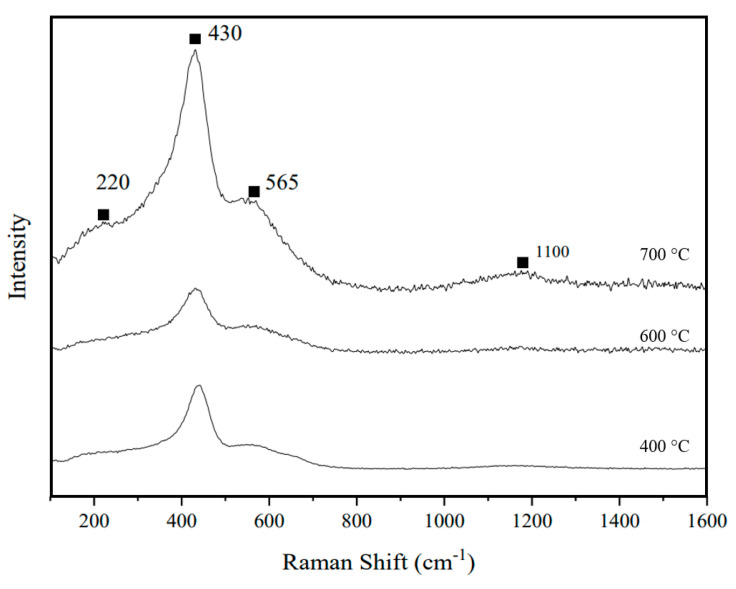
Raman spectroscopy for S2 calcined at 700 °C.

Figure 11 shows the obtained Raman spectroscopy for S_2_, which was used in the fabrication of a symmetrical cell for an electrochemical study. The peak around 450 cm^−1^ indicated the presence of an F_2_g mode in the cubic fluorite structure, which was expected for GDC. It enhanced the XRD findings, which also indicated a cubic structure within the lattice parameters (1 1 1) [54]. The F_2_g mode arises from the oxygen’s stretching vibration occurring within the crystal lattice [55]. A wider peak ranging from 540 cm^−1^ to 600 cm^−1^ indicated the existence of oxygen vacancies, possibly resulting from doped ceria interacting with metals like Co and Fe, leading to additional oxygen vacancies [56,57]. For pure cobalt oxide, Raman spectra exhibited a broad peak between 450 cm^−1^ and 650 cm^−1^, which can be observed to an extent in Figure 11. This observation supports the presence of cobalt oxide [58]. FeO, CoO, and their amalgamation into a crystal lattice are recognized for their cubic structure, exhibiting peaks at 220 cm^−1^ and within the range of 500 to 550 cm^−1^, respectively [59,60]. The Raman spectra aligned with the FT-IR spectra, showing a small peak at 1100 cm^−1^. This peak has been associated with the O-H bending function group [61]. Water or moisture was anticipated to be present as an impurity in the sample because of its contact with the surrounding atmospheric air. Nevertheless, the minimal intensity indicated that only insignificant traces of water persisted after the powder had been dried and calcined. We expected to observe the G-band for FeO around 580 cm^−1^ to 650 cm^−1^; for CoO, the G-band was observed around 660 cm^−1^ to 690 cm^−1^. These bands correspond to the stretching vibrations of the Fe-O and Co-O bonds in the oxide lattice, which were observed in the Raman spectra. Additionally, FeO and CoO did not show a prominent D-band in the Raman spectra, indicating a crystalline structure with minimal defect-induced modes. The peak at 360 cm^−1^, corresponding to cubic Gd203, and its absence in the Raman spectrometer provided additional confirmation that GDC had indeed formed. GDC exhibited a G-band around 460 cm^−1^ to 480 cm^−1^. This band is associated with the vibrational modes of the Ce-O bonds in the ceria lattice, which can be observed in the spectra. The GDC may also exhibit a weak D-band around 1000 cm^−1^ to 1100 cm^−1^, which can be attributed to structural defects or disorder induced by the doping process or synthesis conditions, which can be seen for sample S2 calcined at 700 °C.

**Figure 12:**
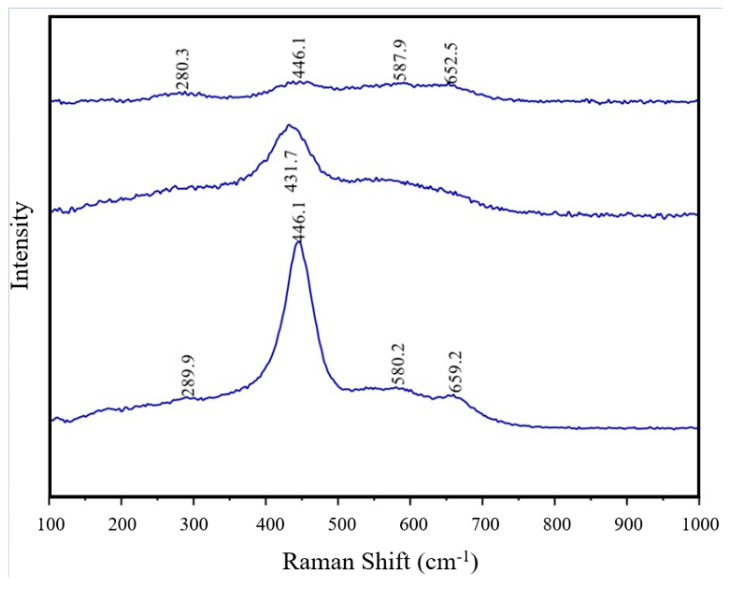
Raman spectra of S5 calcined at 700 °C.

Figure 12 represents the Raman spectra of S5 after calcination at 700 °C in air. The presence of a single distinct peak confirmed the existence of a single crystal phase encompassing FeO, COO, and GDC in the sample. The sharp peak, located around 440 cm^−1^, was associated with the GDC [62]. Furthermore, the absence of a peak at 360 cm^−1^, corresponding to cubic Gd_2_0_3_ [63], provided additional confirmation that GDC had indeed formed. The main peak observed could be associated with CeO_2_, which is characterised by a peak around 460 cm^−1^. This relates to the symmetry in the cubic phase [64]. The strong main peak could be attributed to the presence of cubic FeO and CoO, with peaks at 450 cm^−1^ [65] and 468 cm^−1^ [66], respectively. FeO and CoO were also detected in the weak peaks around 655 cm^−1^, with an associated FeO peak at 650 cm [65] and a CoO peak around 670 cm^−1^ [66]. The slight shift from the expected values could be explained by the incorporation of Gd^3+^ with CeO2 due to the size discrepancy between the Gd^3+^ and Ce^4+^ ions. This could also account for the weak peak at around 580 cm^−1^, which arose from the oxygen vacancies resulting from the replacement of Ce^4+^ ions with Gd^3+^ ions during doping [48]. In the same manner, the addition of FeO and CoO could cause similar shifting effects.

### 3.5. Analysis of Morphology and (EDX/EDS)

**Figure 13:**
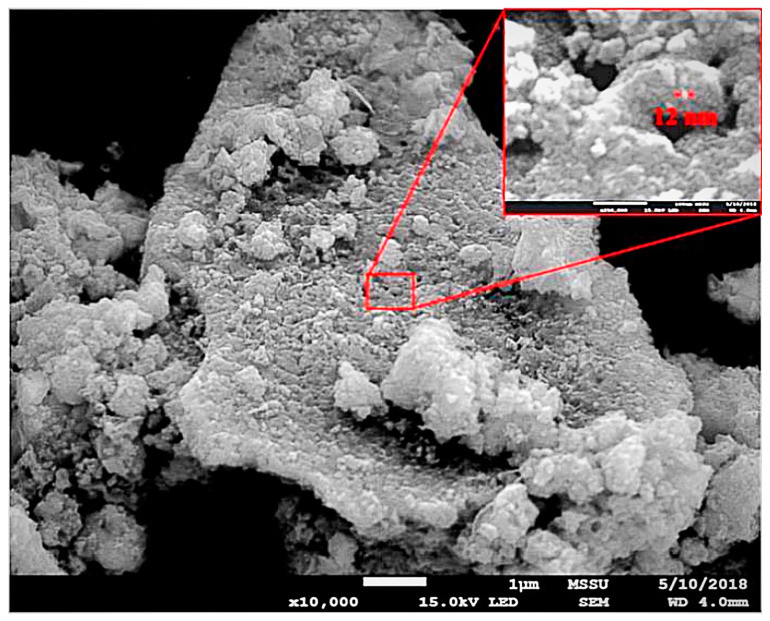
SEM images of S_2_ calcined at 700 °C.

Figure 13 shows the SEM images of S2 after calcination at 700 °C. The average particle sizes displayed a range of 12 to 15 nm. ImageJ software (accessed in September 2021) was utilised to randomly select 20 particles for analysis. These results were consistent with the XRD findings, which indicated that the 10 nm crystals underwent agglomeration to form larger particles, as anticipated. After the samples were subjected to calcination, it was hypothesised that the chemical composition of the samples could fall into three potential cases: case 1, FeO and CoO; case2, Co_2_O_3_ and Fe_2_O_3_; or a combination resulting in case 3, Co_3_O_4_ and Fe_3_O_4_.

For instance, the potential compositions of the end product for S2, intended for cell manufacturing, were given as Ce_0.1773_Gd_0.0443_Co_0.39_Fe_0.39_O_1.199_, Ce_0.1773_Gd_0.0443_Co_0.39_Fe_0.39_O_1.589_ and Ce_0.1773_Gd_0.0443_Co_0.39_Fe_0.39_O_1.459_, respectively. To confirm the chemical composition of the resulting anode powder, an EDX analysis was conducted on powder samples calcined at 400 °C, 600 °C, and 700 °C. The interpretation of the composition was based on the average atomic percentage content, as presented in Table 6. The identification of silicon in the EDX analysis implied that contamination may have been introduced at the time of drying, specifically when the sample was removed from the bulb flask, and small amounts of aluminium, phosphorus, and copper were found. Therefore, these elements were considered impurities from the materials used and were not included in the calculations. The samples were found to contain varying amounts of carbon. As expected, the samples that underwent a higher calcination temperature of 700 °C had significantly less carbon content. A contrast between the 4.11 atoms in % carbon content in the 700 °C sample and the approximately 27 atoms in % carbon content in the 400 °C and 600 °C samples indicated that the changes implemented in the calcination process for the powders utilised in cell production were successful in reducing carbon levels in the samples. The trace contaminants and carbon were not considered in confirming the composition, and the atom percentages were recalculated based only on the relevant elements, as seen in Table 6. The results showed that case 1 closely matched the EDX results, suggesting that the final product had a chemical composition of Ce_0.1773_Gd_0.0443_Co_0.39_Fe_0.39_O_1.199_, with the presence of FeO and CoO in the sample. It was unexpected to find that the samples did not contain Fe_2_O_3_ and CoO after calcination, contrary to what was initially assumed. This could be because the Fe_2_O_3_ was reduced to FeO due to the presence of carbon monoxide. It is possible that the oxygen in the Fe_2_O_3_ was removed to form CO_2_.

**Figure 14:**
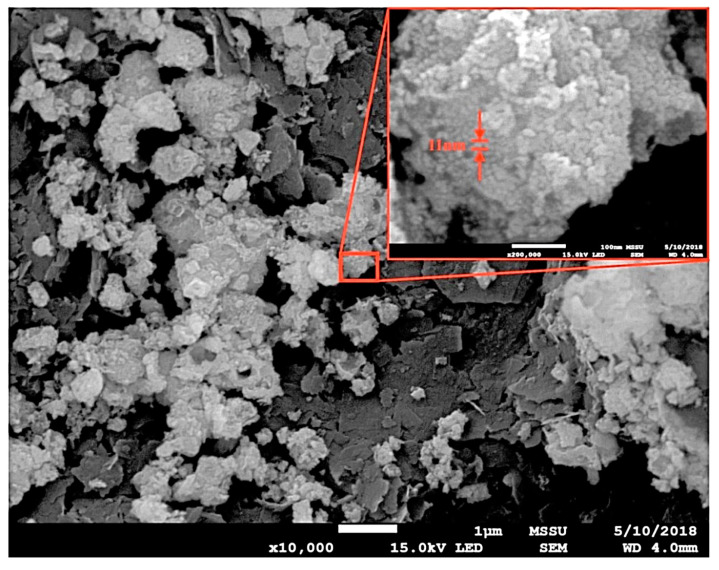
SEM image of S_5_ calcined at 700 °C.

Figure 14 shows the SEM image of S5 calcined at 700 °C, revealing a particle size of 11 nm. This aligned with the mean crystalline size calculated from the XRD results, as shown in Table 7, confirming that the material synthesis resulted in the production of a nanopowder.

As anticipated from the original XRD results, the EDX data in Table 7 confirmed the presence of a higher amount of carbon at low calcination temperatures. This could be attributed to an insufficient temperature or the lack of oxygen during the initial calcination stages, although the chosen calcination method yielded a low carbon content. The samples also included trace amounts of aluminium, phosphorus, and copper. It is believed that these elements were introduced during the EDX analysis, contributing to such negligible atom percentages that these contaminations were not representative of the synthesis method of the powders. Therefore, this method was considered to be appropriate.

### 3.6. Electrical Conductivity

**Figure 15:**
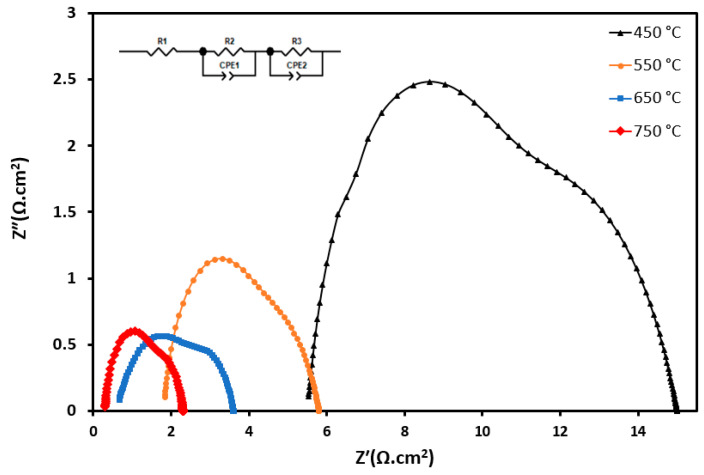
Impedance spectra of the symmetric cell using S_2_ as the electrode and GDC as the electrolyte tested in H_2_ from 450 to 750 °C.

Figure 15 displays the Nyquist plot of the impedance spectra for the symmetric anode cells that were evaluated at temperatures from 450 °C to 650 °C in air. The arc’s size reflects the extent of the polarisation losses, and the Nyquist plots were additionally examined utilising the equivalent circuit model shown in Figure 15. However, as the spectra suggest, two major processes can be identified at different temperatures; the spectra recorded at 450 °C slightly show three semicircles. The high-frequency arc seems to be overlapped and disappear as the temperature increases. These semicircles may be ascribed to the presence of grain boundaries, grain impedance, and the interfaces between the electrolyte and the electrode (current collector). In this work, the equivalent circuit fit was obtained by implementing a model with two constant phase elements, each in parallel with a resistor, as inserted in Figure 15. At higher frequencies, the ohmic resistance can be identified by examining the point where the impedance semicircle intersects the (Z′) in the Nyquist plot, as shown in Table 8.

The values for the area-specific resistance (ASR) were determined by analysing the intercepts on the real axis, and a summary of these findings can be found in Table 8. The ASR decreased as the temperature increased, which is the identification of semiconductors, and there was no Warburg impedance observed in these EIS data.

As the temperature increased, the ionic conductivity of the electrolyte increased, resulting in a decrease in ohmic resistance. This is because higher temperatures generally enhance the mobility of ions in the electrolyte leading to a lower ohmic resistance (Rs) as the temperature increases, as shown in Table 8. The charge transfer resistance, as shown in Table 8 by Rp1 and Rp2, was associated with the electrochemical reactions at the electrode/electrolyte interfaces. As the temperature increased, several factors such as reaction kinetics, electrode activity, and diffusion rates improved, leading to a decrease in charge transfer resistance, which is represented by the diameter of the semicircle in the Nyquist plot of the EIS data (Figure 15). As the temperature rose, the rate of the electrochemical reactions kinetics increases, leading to a reduction in the resistance associated with relevant reactions. Additionally, increasing the temperature often improves the catalytic activity of the electrodes, lowering the resistance and facilitating charge transfer. Similarly, at elevated temperatures, the diffusion of reactants to TPB and products from TPB is faster leading to a lower charge transfer resistance. In this report no Warburg impedance was observed but increasing the temperature led to a decrease in the Warburg impedance, as at higher temperature, diffusion increases due to improved mass transfer.

Overall, the decrease in ohmic and charge transfer resistances as the temperature was elevated led to an improvement in the electrochemical performance of the SOFC due to the faster and more efficient reactions occurring at the reaction sites.

**Figure 16:**
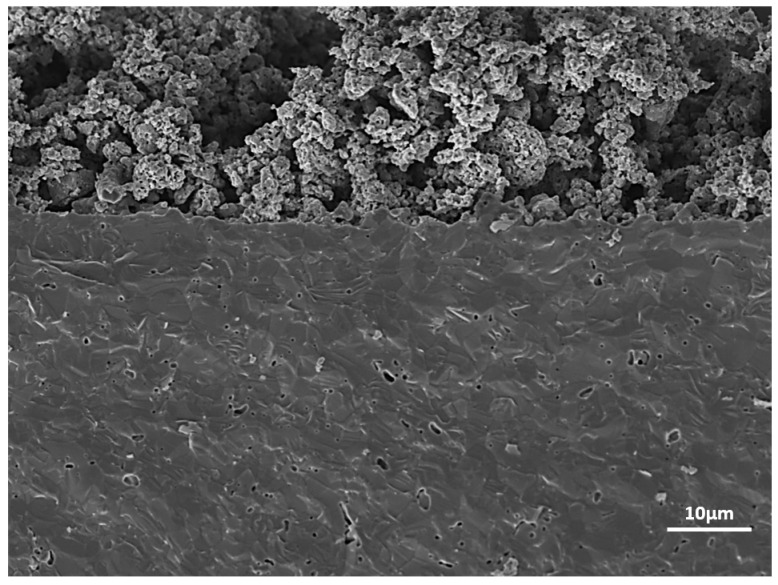
SEM images of the tested cell: cross-sectional microstructures of the Fe/Co-GDCǀGDCǀFe/Co-GDC symmetrical cell with S_2_.

Figure 16 illustrates the cross-sectional SEM image of the fabricated symmetrical cell, showing the Fe/Co-GDC/GDC bilayer for the electrolyte-supported cell. The effective connection between the electrolyte and the anode layer is clearly visible. The dense electrolyte layer and porous anode layer are shown to have a thickness of almost 400–500 µm and 20–30 μm, respectively. The anode’s porous structure facilitates the efficient exchange of gaseous reactants and products at the triple-phase boundary (TPB). The good interconnection between the anode and electrolyte is mainly due to the homogeneity and nano-size anode material used to fabricate the cell.

**Figure 17:**
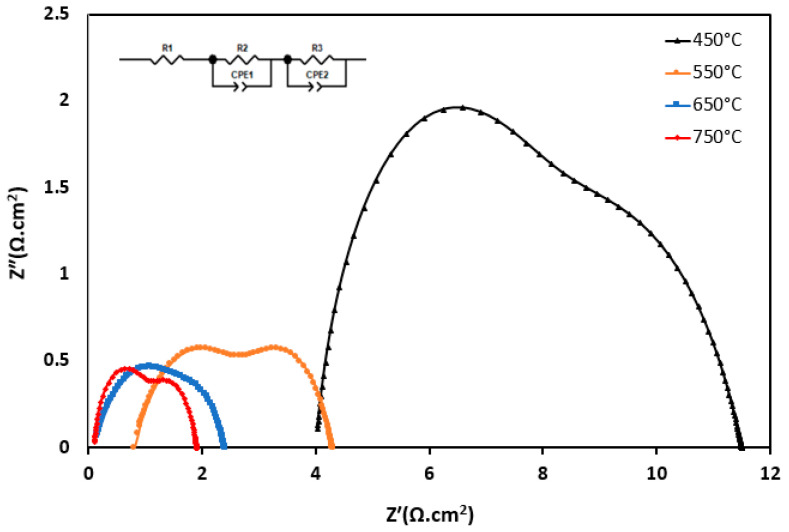
Impedance spectra of the symmetric cell using S_5_ as the electrode and GDC in H_2_ from 450 to 750 °C.

A Nyquist plot from the symmetric cell for S_5_ is shown in Figure 17. The electrode reactions consist of two primary components: a lower-frequency arc and a higher-frequency arc. Based on the literature [67], the low-frequency arc is affected by microstructural features and temperature, while the high-frequency arc contributes to the gas composition. As a result, the high-frequency arc at the electrode–electrolyte boundary indicates both double-layer capacitance and ion transfer resistance; the low-frequency arc, on the other hand, indicates that the electrode kinetics are affected by the composition of the gas. Therefore, the low-frequency arc in all samples is likely to remain relatively constant, while the high-frequency arc decreases as the particle size decreases. Thus, S_5_, as the sample with the smallest particle size, was chosen to be tested for the electrochemical study. The polarisation resistance for the fabricated cell is presented in an *R*_p_ value of around 1, and 0.4 Ω cm^2^ was reported for the anodes of Ni-YSZ and Ni–GDC, respectively [68,69] in a 650 °C environment with humidified H2; the polarisation resistance of S5 registered at 0.75 Ω cm^2^ at that temperature. The Rp in this work was less than that of the Rp in the Ni-GDC anode material, which could be due to the type of support used in these works (the cell from the literature cell was anode-supported, while the cell in this work was electrolyte-supported). Another factor that can be modified to improve the Rp for the fabricated cell is the composition of the gas used as the feed (in the mentioned literature, wet hydrogen was used, while in our work, the cells were exposed to dry hydrogen). Despite a relatively high Rp, the findings suggest that CoxFe1-xGDC demonstrates elevated levels of activity in the electrochemical oxidation of hydrogen. Table 9 shows the polarisation resistance at various temperatures.

**Figure 18:**
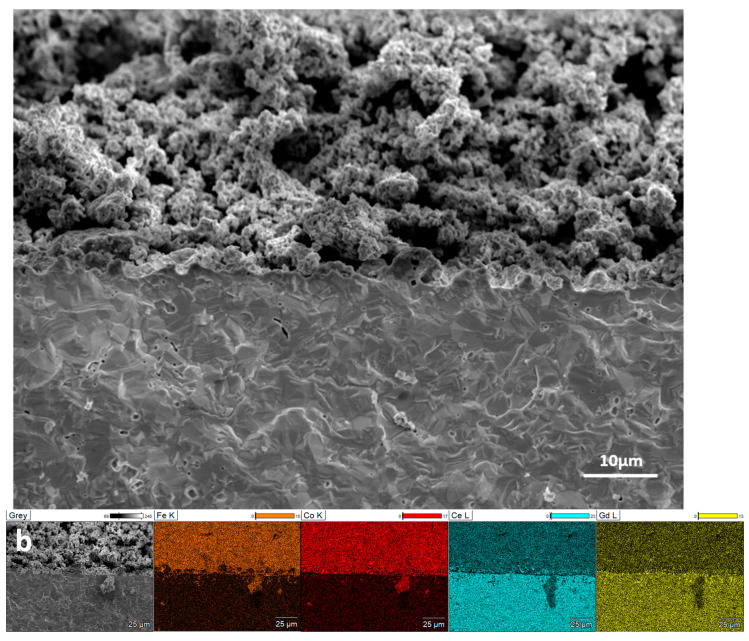
SEM images (**a**) and EDX of the tested cell (**b**): cross-sectional microstructures of Fe/Co-GDCǀGDCǀFe/Co-GDC symmetrical cell with S_5_.

In Figure 18a, the tested single cell’s cross-sectional SEM images apparently indicate that the symmetrical cell was constructed by enclosing a GDC electrolyte with porous S2 composite anode material. The thickness of the electrode and electrolyte was ∼20–30 μm and ∼400–500 μm, respectively. The SEM image indicates that the GDC electrolyte was relatively dense, crack-free, and had some pinholes, which can be why the resistance was lower than expected. The achievement of a strong bond between the electrolyte and the electrodes could be attributed to the utilisation of nano-sized anode material. This successful adhesion played a significant role in reducing the single cell’s ohmic resistance. The uniformity of the anode’s composition is apparent in the EDX results from Figure 18b. The relatively high porosity of the anode layer facilitated the gas diffusion to and from electrode–electrolyte interconnections.

## 4. Conclusions

CoxFe1-xO/GDC nano-crystalline powders of varying compositions (x = 0.2, 0.5, 0.8) with volume ratios of metal/GDC of 50/50 and 40/60 were synthesised using a green co-precipitation method. The EDX results confirmed the formation of material with the expected composition, and XRD, SEM, Raman, and FTIR analyses were employed to study the formation of metal oxides at different temperatures and to confirm the formation of nano-sized particles. However, as the calcination temperature increased, more carbon-free products could be obtained, but the particle size increased, which is not desirable for the anode material in SOFC fabrication. EIS was used to further investigate the effect of the GDC load on the electrochemical properties of the synthesised material. For that purpose, Co_x_Fe_1-x_O/GDC (x = 0.2) and bimetal/GDC 50/50 V% (S2), and Co_x_Fe_1-x_O/GDC (x = 0.2) and bimetal/GDC 50/60 V% (S5) were chosen for the fabrication of a symmetrical cell. It was found that as the GDC content increased, the resistance decreased, and Rpt values of 1.53 and 1.15 Ω were observed for a single electrode using S2 at 650 and 750 °C, while Rpt values of 0.75 and 0.67 Ω were achieved for S5 at the same temperature. It could be due to the better interconnection between the electrolyte and anode film, which was clearly apparent in the cell’s SEM images.

## Figures and Tables

**Table 1:** The crystal size of the samples S1, S2, and S3 before and after calcination.

Sample ID	Crystal Size (nm)			
	Before Calcination	After Calcination		
		400 °C	600 °C	700 °C
S_1_	81.15	4.303	7.097	-
S_2_	70.87	4.883	5.307	10.85
S_3_	79.82	6.269	7.838	-

**Table 2:** Lattice parameters at different calcination temperature.

Sample ID	Calcination Temperature (°C)	Theta (Degree)	Lattice Spacing, d (nm)	Miller Indices			Lattice Parameter (Å)
				h	k	l	
S_1_	400	17.52	0.2560	1	1	1	4.434
S_2_	400	18.07	0.2486	1	1	1	4.305
S_3_	400	18.41	0.2441	1	1	1	4.228
S_1_	600	17.75	0.2529	1	1	1	4.381
S_2_	600	18.09	0.2483	1	1	1	4.300
S_3_	600	18.36	0.2448	1	1	1	4.239
S_2_	700	17.78	0.2524	1	1	1	4.372

**Table 3:** Miller indices and corresponding 2θ for ammonium nitrate.

Ammonium Nitrate [45]	
h k l	2θ (deg)
1 0 0	17.9
0 1 1	22.4
1 1 0	24.3
1 1 1	28.9
0 2 0	32.9
1 0 2	36.1
1 1 2	39.9

**Table 4:** Miller indices and corresponding 2θ for Fe (II) oxide, Co (II) oxide, Ce (IV) oxide, and Gd (III) oxide.

FeO [46]		CoO [47]	
**h k l**	**2** **θ** **(deg)**	**h k l**	**2** **θ** **(deg)**
1 1 1	36.2	1 1 1	36.5
2 0 0	42.0	2 0 0	42.5
2 2 0	61.1	2 2 0	61.6
2 2 2	76.5	2 2 2	77.7
CeO_2_ [48]		Gd_2_O_3_ [49]	
**h k l**	**2** **θ** **(deg)**	**h k l**	**2** **θ** **(deg)**
1 1 1	28.6	1 1 1	28.3
2 0 0	33.1	2 0 0	32.9
2 2 0	47.5	2 2 0	47.3
2 2 2	56.4	2 2 2	56.1

**Table 5:** Estimated crystalline size, lattice parameter, and lattice contraction of all samples after calcination.

Sample ID	Calcination Temperature (°C)	Mean Crystalline Size (nm)	Lattice Parameter (nm)	Lattice Contraction (%)
S1	400	4.1	0.540	0.33
	600	7.1	0.538	0.70
S2	400	3.6	0.531	1.99
	600	4.6	0.533	1.62
	700	10.7	0.537	0.89
S3	400	4.5	0.526	2.92
	600	6.3	0.526	2.92

**Table 6:** EDX elemental analysis of sample S2 after calcination.

Element	Calcination Temperature (°C) for S2				Cases	
	400	600	700	Case1 Atom%	Case2 Atom%	Case3 Atom%
	Average Atom%					
O-K	49.9%	53.9%	43.8%	54.5%	61.4%	59.3%
Fe-K	19.8%	17.6%	20.6%	17.7%	15.0%	15.8%
Co-K	19.2%	18.7%	22.6%	17.7%	15.0%	15.8%
Ce-L	8.3%	7.5%	9.2%	8.1%	6.8%	7.2%
Gd-L	2.8%	2.3%	2.8%	2.0%	1.7%	1.8%

**Table 7:** EDX elemental analysis data for S5 at different calcination temperatures.

Calcination Temperature (°C)	C-K	O-K	Al-K	P-K	Fe-K	Co-K	Cu-K	Ce-K	Gd-K
400	32.6	35.3	0.2	0.2	13.2	12.8	0.6	3.8	1.2
600	26.1	39.3	0.4	0.1	13.7	15.4	NA	3.8	1.2
700	6.8	45.2	0.4	NA	19.2	20.8	NA	5.8	1.9

**Table 8:** Area-specific resistances extracted from EIS data for single electrode cell S_2_.

Temperatures	Sample ID	R_s_	R_P1_	R_P2_	R_pt_	Area-Specific Resistance (ASR) (Ohm cm^2^) (R_Pt_)
450 °C	S_2_	5.53	7.37	4.56	5.967	1.49
550 °C	S_2_	1.83	2.72	2.27	4.49	0.63
650 °C	S_2_	0.66	1.84	1.22	1.53	0.38
750 °C	S_2_	0.31	1.29	1	1.15	0.29

**Table 9:** Fitted equivalent circuit polarisation resistances for symmetrical cell.

Temperatures	Sample ID	Rs	R_P1_	R_P2_	R_pt_	Area-Specific Resistance (ASR) (Ohm cm) (R_Pt_)
450 °C	S5	4.03	4.92	4.02	4.47	1.12
550 °C	S5	0.81	2.18	1.65	1.92	0.48
650 °C	S5	0.15	0.91	0.60	0.75	0.19
750 °C	S5	0.12	0.84	0.49	0.67	0.17

## Data Availability

The original contributions presented in the study are included in the article, further inquiries can be directed to the corresponding authors.
